# Artificial Gravity as a Countermeasure to the Cardiovascular Deconditioning of Spaceflight: Gender Perspectives

**DOI:** 10.3389/fphys.2018.00716

**Published:** 2018-07-06

**Authors:** Joyce M. Evans, Charles F. Knapp, Nandu Goswami

**Affiliations:** ^1^Department of Biomedical Engineering, University of Kentucky, Lexington, KY, United States; ^2^Physiology, Otto Loewi Research Center for Vascular Biology, Immunology and Inflammation, Medical University of Graz, Graz, Austria

**Keywords:** orthostatic intolerance, microgravity, aging, spaceflight simulations, falls

## Abstract

Space flight-induced physiological deconditioning resulting from decreased gravitational input, decreased plasma volume, and disruption of regulatory mechanisms is a significant problem in returning astronauts as well as in normal aging. Here we review effects of a promising countermeasure on cardiovascular systems of healthy men and women undergoing Earth-based models of space-flight. This countermeasure is produced by a centrifuge and called artificial gravity (AG). Numerous studies have determined that AG improves orthostatic tolerance (as assessed by various protocols) of healthy ambulatory men, of men deconditioned by bed rest or by immersion (both wet and dry) and, in one case, following spaceflight. Although a few studies of healthy, ambulatory women and one study of women deconditioned by furosemide, have reported improvement of orthostatic tolerance following exposure to AG, studies of bed-rested women exposed to AG have not been conducted. However, in ambulatory, normovolemic subjects, AG training was more effective in men than women and more effective in subjects who exercised during AG than in those who passively rode the centrifuge. Acute exposure to an AG protocol, individualized to provide a common stimulus to each person, also improved orthostatic tolerance of normovolemic men and women and of furosemide-deconditioned men and women. Again, men’s tolerance was more improved than women’s. In both men and women, exposure to AG increased stroke volume, so greater improvement in men vs. women was due in part to their different vascular responses to AG. Following AG exposure, resting blood pressure (via decreased vascular resistance) decreased in men but not women, indicating an increase in men’s vascular reserve. Finally, in addition to counteracting space flight deconditioning, improved orthostatic tolerance through AG-induced improvement of stroke volume could benefit aging men and women on Earth.

## Cardiovascular Responses to Space Flight and Similarities to Aging

A myriad of cardiovascular effects develop during space flight, including an immediate shift of fluid headward and a decrease in central venous pressure (even though transmural central venous pressure increases and therefore venous return of blood to the heart is enhanced) (Buckey et al., 1996a; Levine et al., 2002; Kaderka, 2010; Norsk, 2014). On a longer time scale, astronauts develop a loss of ventricular mass (cardiac atrophy) (Perhonen et al., 2001; Dorfman et al., 2007, 2008), decreased sensitivity of the carotid-cardiac (vagal) baroreflex (Convertino, 2002; Norsk, 2014) and a greater responsiveness of sympathetic neural activity to inflight simulations of standing (Ertl et al., 2002). Overall, the net effect of time spent in space is manifested by decreasing blood pressure and elevation of cardiac output throughout flight implicating peripheral vasodilation as a major body response that may drive the reduction of plasma volume and associated cardiovascular effects. Conundrums exist in the elevation of cardiac output in the face of cardiac atrophy and in the fact that muscle sympathetic nerve activity (MSNA) increases during spaceflight despite, or perhaps to counteract, peripheral vasodilation (Ertl et al., 2002; Levine et al., 2002; Norsk, 2014; Norsk et al., 2015).

Upon return from space missions, cardiovascular effects have been a concern from the time of early Mercury flights when two astronauts were found to have lost tolerance for standing even after a short time spent in weightlessness (Kaderka, 2010). To date, the most persistent post-flight problem has been orthostatic intolerance (OI), as demonstrated in up to 64% of returning astronauts (Buckey et al., 1996b; Platts et al., 2014). Major cardiovascular conditions that present upon return from space flight include increased hematocrit, decreased plasma volume, decreased aerobic capacity, cardiac atrophy, decreased norepinephrine, and decreased vascular responsiveness in response to standing, (even though directly measured MSNA is increased during space flight) (Perhonen et al., 2001; Levine et al., 2002; Kaderka, 2010; Norsk, 2014).

Spaceflight and aging are associated with similar kinds of physiological deconditioning. For example, microgravity during spaceflight has been shown to influence cardiovascular function, cerebral autoregulation, and musculoskeletal function, in a manner that leads to OI upon return to Earth (Blaber et al., 2013; Goswami et al., 2013). Similarly, aging is associated with deterioration of cardiovascular and musculoskeletal systems, which predisposes older persons to dizziness upon standing and/or OI, which can lead to falls and falls-related injuries, and often hospitalization (Blain et al., 2016; Bousquet et al., 2017). Specifically, current scientific knowledge regarding OI and how it comes about provides a framework for understanding (patho-) physiological concepts of cardiovascular (in-) stability in bed rest-confined senior citizens or those on multiple medications (polypharmacy) (Goswami et al., 2017).

Furthermore, since bed rest is used as a model to study effects of spaceflight de-conditioning (Cvirn et al., 2015; Goswami et al., 2015a; O’Shea et al., 2015; Waha et al., 2015) and hospitalized older persons spend a large part of their time in bed, the de-conditioning effects of bed rest confinement on physiological functions and its parallels with spaceflight deconditioning can be exploited to understand and combat both variations of de-conditioning. This knowledge is important as de-conditioning due to bed confinement in older persons can lead to a (downward) spiral of increasing frailty, OI, falls, and fall-related injury.

Integration of knowledge regarding deconditioning due to reduced gravitational stress in space, and bed rest-induced deconditioning promotes a comprehensive approach that can incorporate nutritional aspects, muscle strength, and function (Gao et al., 2018), cardiovascular (de-) conditioning, and cardio-postural interactions (Goswami et al., 2013). The impact of such integration can provide new insights and lead to methods of value for both space medicine and geriatrics (Geriatrics meets Spaceflight!) (Goswami, 2017). Finally, as astronauts in space spend substantial amounts of time carrying out exercise training to counteract the microgravity-induced de-conditioning – and to counteract OI on return to Earth-, it is logical to suggest some of these interventions for bed-confined older persons.

### Gender Differences

Cardiovascular gender/sex differences noted upon return to Earth, indicate that women are more susceptible to post-flight OI (Fritsch-Yelle et al., 1994; Harm et al., 2001; Waters et al., 2002; Platts et al., 2014) while visual impairment intracranial pressure syndrome appears to be more severe in men (Mark et al., 2014; Platts et al., 2014). In searching for mechanisms responsible for reduced orthostatic tolerance following spaceflight, studies have determined that women demonstrate a greater loss of plasma volume (Waters et al., 2002; Platts et al., 2014), a greater decrease in baroreflex control of heart rate (Fritsch-Yelle et al., 1994; Waters et al., 2002) and an hypoadrenergic responsiveness to orthostatic stress (Waters et al., 2002). Several reports also note that, on Earth, women typically respond to stress with increased heart rate, while men respond with increased vascular resistance (Ludwig et al., 1987; Evans et al., 2001; Arzeno et al., 2013; Mark et al., 2014). The lower vasoconstrictive reserve of women in the Convertino study came in spite of steeper increases in peripheral vascular resistance accompanied by enhanced epinephrine and diminished norepinephrine responses at presyncope, leading the authors to conclude that women clearly demonstrated less effective responsiveness of mechanisms that contribute to blood pressure regulation during orthostatic stress (Convertino, 1998). These results are reinforced by those seen in women’s response to both standing (Waters et al., 2002; Meck et al., 2004) and lower body negative pressure (LBNP) stresses (White et al., 1996; Frey and Hoffler, 1998). Finally, coherence between diastolic blood pressure and MSNA is lower in women compared to men at rest and in response to increasing LBNP (Yang et al., 2012).

Recently, a study of post-flight carotid artery stiffness and associated blood biomarkers indicated that, after 6 months in space, carotid artery stiffness and insulin resistance were increased in that group of astronauts with sex differences noted in pulse transit time, insulin resistance, plasma renin, and aldosterone (Hughson et al., 2016). Overall, the latter study indicated that the significant gender differences from this small group of astronauts who had spent 6 months on the International Space Station, would require additional research to firmly establish gender-specific differences in these important metabolic and vascular remodeling variables (Hughson et al., 2016). Autoregulatory differences in cerebral blood flow may also contribute to greater Ol in women compared to men. Cerebral flow regulation in astronauts who failed a post-flight 10 min stand test was characterized by higher cerebral vasodilation with a significant sex interaction in response to standing: five of eight non-finishers were female while 17 of 19 finishers were male (Blaber et al., 2011). Harm et al. (2001) carried out a compilation of statistics from 140 males and 25 females with respect to occurrence of Ol following 5–16 days of spaceflight and reported that 7% of males and 28% of females developed Ol, similar to that reported by Blaber et al. (2011).

One study established foundations for basic gender differences by using ganglionic blockade to examine reflex responses to vasoactive drugs (Christou et al., 2005). In that study the authors determined that, in the presence of ganglionic blockade, men and women displayed equal increases in blood pressure in response to an alpha 1 agonist. However, before blockade, the women’s response was significantly greater than the men’s response, clearly establishing that baroreflex buffering of blood pressure by reflex lowering of heart rate was not as robust in reflexive women as it was in reflexive men. The same study also established that women’s vasopressin response to the ganglionic blocker was significantly smaller than that of men’s, thereby indicating that women’s secretion of that hormone to support blood pressure was not as strong as men’s (Christou et al., 2005).

## Cardiovascular Responses to Ground Based Simulations of Space Flight

### Simulations of Spaceflight

Certain criteria from spaceflight (headward shift of fluid) and return from spaceflight (increased hematocrit and Ol) led to the choice of head down bed rest (HDBR) and immersion (both wet and dry) as the Earth-based protocols that best simulate cardiovascular responses to actual spaceflight. As a general rule, HDBR and dry immersion are used to model long term effects of spaceflight while neck-high immersion in thermo-neutral water is used to model short term effects (Norsk, 2014).

Questions arise concerning the suitability of different models of spaceflight to provoke responses comparable to those seen post-flight. Accompanying this concern are: questions of the appropriate amount of time over which to apply such models in order to induce certain deconditioning effects; the appropriate testing of countermeasures to combat this cardiovascular deconditioning; and the most appropriate model to produce the gender effects seen in actual spaceflight.

### Similarities and Differences Between Spaceflight Simulations

At rest, similarities between HDBR and wet and dry immersion include headward fluid shift, decreased plasma volume, increased venous distensibility, decreased heart muscle strength, and muscle volume (cardiac atrophy) as well as impaired carotid-cardiac control of heart rate that occurs on a quicker time scale in response to wet or dry immersion than to HDBR (Fortney, 1991; Levine et al., 1997; Perhonen et al., 2001; Norsk, 2014). Differences between water immersion and HDBR include a larger shift of fluid to the chest accompanied by a greater increase in heart size with immersion and a larger shift of fluid to the head with HDBR. In addition, blood pressure decreases acutely in response to HDBR but not to water immersion (Norsk, 2014).

### Similarities and Differences Between Spaceflight Simulations and Spaceflight

Similarities between immersion to the neck in thermo-neutral water (WI) and spaceflight are the immediate shifting of fluid to the thorax resulting in the acute increase in cardiac preload accompanied by intravascular absorption of interstitial fluid, two mechanisms that seem to dominate the early response to spaceflight (Norsk, 2014).

Similarities of HDBR to spaceflight include a 10–15% decrease in plasma volume accompanied by diminished cardiac performance and baroreflex sensitivity that mirror those of spaceflight (Levine et al., 1997; Dorfman et al., 2007; Pavy-Le Traon et al., 2007). In addition, HDBR subjects experience a rapid decline in aerobic capacity followed by a further decline at a slower rate, impaired vascular reflexes, and altered myocardial mechanics, similar to those observed during spaceflight (Levine et al., 1997; Dorfman et al., 2007; Hargens et al., 2013). In terms of autonomic behavior, HDBR has been shown to result in decreased baroreflex sensitivity in control of heart rate and augmented sensitivity in control of MSNA, also similar to that of spaceflight (Arzeno et al., 2013). Finally, again similar to spaceflight, both cardiac atrophy and MSNA increase across the course of HDBR while the decrease in plasma volume plateaus (Levine et al., 1997; Kamiya et al., 2000; Perhonen et al., 2001; Dorfman et al., 2007; Arzeno et al., 2013; Norsk, 2014).

One critically important difference between HDBR and space flight, is the effect of gravity acting on the intra thoracic pressure of HDBR subjects when compared to the dramatic release of thoracic compression (due to lifting of the weight of the chest walls) in space. Another important difference is MSNA, which, although increasing across both space flight and HDBR, is at higher levels during spaceflight (Norsk, 2014). The effects of MSNA on vascular resistance and thoracic compression on venous return are important and need to be considered when interpreting results of HDBR studies (Norsk, 2014).

### Gender Similarities and Differences in Simulations of Space Flight

#### Similarities

Cardiac atrophy, observed with both echocardiography and magnetic resonance (MR), indicated a loss of left and right ventricular mass that increased similarly in men and women during 60 days of sedentary, HDBR (Dorfman et al., 2007). Studies have also determined that exercise during HDBR attenuated the loss of cardiac mass or left ventricular compliance to approximately the same degree in both men and women (Arbab-Zadeh et al., 2004; Dorfman et al., 2007). Similarly, a 60 day HDBR study found that some differences in autonomic control (men’s tendency toward sympathetic activation and women’s parasympathetic dominance in terms of heart rate effect on blood pressure regulation) were preserved even though both parasympathetic modulation and baroreflex sensitivity decreased across bed rest (Arzeno et al., 2013). Greater parasympathetic dominance in healthy, ambulatory women, and greater sympathetic dominance in ambulatory men were previously reported by Frey and Hoffler (1998) and also by our laboratory (Evans et al., 2001). Even short term (4 h) HDBR indicates that both men and women demonstrate similar lowering of blood volume, central venous pressure, forearm vascular resistance, and norepinephrine and higher heart rate and greater loss of stroke volume during LBNP compared to 4 h of seated rest (Edgell et al., 2012).

#### Differences

A study comparing orthostatic tolerance before and after 6 h of water immersion with that determined before and after 6 h of HDBR, found that men’s orthostatic tolerance limit (OTL) was greater than women’s in all cases, and the decrease in tolerance was greater in women than in men after HDBR but not after water immersion (Hordinsky et al., 1981). Two important measures of autonomic function, determined in the Arzeno study, indicated that women’s baroreflex sensitivity decreased more than men’s in response to HDBR and the group’s decrease in systolic and diastolic blood pressure over 60 days of HDBR was due to men, while women maintained blood pressure over the course of the study (Arzeno et al., 2013). Although HDBR-induced decrease in parasympathetic modulation would lead to decreased baroreflex sensitivity, it is unclear what role these changes play in the greater susceptibility to OI and greater loss of plasma volume in female, compared to male, astronauts upon return to Earth (Mark et al., 2014).

A bed rest study by Pavy-Le Trao et al. (2002) showed that, even though there was a slower vasodilatory response to sudden reductions in blood pressure in orthostatically intolerant women, cerebrovascular autoregulation was not impaired in females. However, the large variability in cerebral blood flow responses during HDBR studies, seriously limits the use of HDBR-derived cerebral blood flow data in understanding how cerebral vasculature adapts to microgravity exposure (Blaber et al., 2013).

## Cardiovascular Responses to Artificial Gravity

Artificial gravity (AG) as a countermeasure to physiologic deconditioning of multiple organ systems has long been discussed and proposed (National Research Council, 2011, Chapter 7 of Recapturing a Future for Space Exploration; Shulzhenko, 1992; Vernikos et al., 1996; Vernikos, 1997; Greenleaf et al., 1998; Clement and Pavy-Le Traon, 2004; Evans et al., 2004, 2015; Clement et al., 2016). To date, however, cardiovascular responses to AG applied in the long body axis (to simulate standing on Earth) have come almost exclusively from Earth-based studies in a variety of situations: (1) healthy, ambulatory subjects acutely exposed to hypergravity, (2) healthy, ambulatory subjects before and after a period of AG training, and (3) deconditioned subjects before and after bed rest, HDBR, water immersion, dry immersion, and furosemide-induced simulations of spaceflight. Below, we review what has been learned about the human response to AG in the above environments and seek to determine if gender differences in cardiovascular responses might affect the future of AG as a countermeasure to cardiovascular deconditioning. There are two studies where AG was actually applied to astronauts several times in-flight in order to gather their perception of AG while in space (Benson et al., 1997; Clément et al., 2001). Although those studies did not report actual post-flight cardiovascular responses to gravitational stimulus, later reports (Clement and Pavy-Le Traon, 2004; Clement et al., 2016) stated that none of the four astronauts who underwent AG during that flight exhibited post-flight OI while the three who did not receive AG, did exhibit OI. Although there are a plethora of ground based studies, the need for actual space-based studies focusing on cardiovascular and other system effects of AG is glaring, and has been called out by NASA administrators as a goal for closing critical gaps in the areas of post-flight human performance (National Research Council, 2011, Chapter 7 of Recapturing a Future for Space Exploration; Kaderka, 2010; Mark et al., 2014; Norsk, 2014; Clement et al., 2016).

As glaring as the lack of space-based information from humans who have experienced in-flight AG, is the lack of data from women undergoing AG during bed rest or immersion (wet or dry) studies. Acute responses to AG have been determined from healthy, normovolemic women (Stenger et al., 2007) and from healthy women deconditioned by furosemide (Evans et al., 2015; Zhang et al., 2017). However, there are no AG studies in women deconditioned by HDBR or immersion (wet or dry); the three simulations of space flight considered closest to actual space missions. Therefore, results below will summarize what has been learned from numerous AG studies conducted in healthy men studied in ambulatory conditions and following simulations of space flight, and from the few studies conducted in healthy ambulatory women and the only study of deconditioned (furosemide infusion) women.

### Healthy Ambulatory Subjects

#### Acute Exposure to AG

##### Normovolemic men

Exposure of healthy, ambulatory men and women to AG is a mainstay of pilot testing and training and such testing has also been used in the general population to collect baseline data for commercial spaceflight (Blue et al., 2012). In that study, data were collected from 65 men and 12 women, 22–88 years old, as they were taken to gray-out with the principal finding being that gray-out and peak heart rate were inversely related to age. Another study of 22 men and 25 women determined that risk factors for OI to G stress (on NASA Ames’ centrifuge) included increased height and reduced plasma volume (Ludwig et al., 1987).

In the last 12 years, centrifugation in the long body axis has been used to select subjects for subsequent bed rest studies where AG was to be tested as a countermeasure to subsequent HDBR (Fong et al., 2007). In that study, 5/6 men were tolerant of a +1 Gz load at the heart (+2.5 Gz at the feet) applied for an hour. A similar study designed to evaluate the role of anthropometric factors in determining cardiovascular stability during two bouts of AG found that tolerance correlated positively with body volume and fat free mass. In that study, 8 of the men were classified as high tolerance and two were classified as low tolerance (Opatz et al., 2014).

##### Normovolemic women

In the above studies, women were also tested for their tolerance to AG. Being female was a significant risk factor for OI in the Ludwig study (Ludwig et al., 1987). In the Fong study, only 1/5 women were able to withstand a constant +1 Gz load at the heart for an hour, while in the Opatz study, 6/10 women were tolerant of the two bouts of +2 Gz (Fong et al., 2007; Opatz et al., 2014).

Taken together, results of the latter two studies indicate a higher tolerance in men (13/16) than in women (7/15) for matching AG stresses applied at heart level. Further, the intolerance of women for the constant 1 h, +1G protocol of the Fong study was one of the factors that led to the exclusion of women from a subsequent HDBR/AG study (Stenger et al., 2012).

#### Chronic Exposure to AG

In examining effects of high G training on orthostatic tolerance of men and women, Convertino et al determined that 4 weeks of AG training (three times a week) at ever increasing orthostatic load, increased calf compliance of both men and women but did not remove the lower orthostatic tolerance of women compared to men (Convertino et al., 1998). A study of effects of chronic AG exposure on subjects deconditioned by dry immersion found that intermittent exposure to 0.8–1 Gz during 7 days of immersion prevented the 28% decrease in orthostatic tolerance seen with immersion alone (Vil-Viliams, 1994).

Due to the sparseness of women’s AG studies, the majority of available data in the following section will come from investigations conducted by the authors. Since 1999 we conducted studies of effects of AG training on: ambulatory, acutely deconditioned (furosemide) and bed rest deconditioned men, (Greenleaf et al., 1998; Evans et al., 2004; Stenger et al., 2012; Blaber et al., 2013), and on ambulatory and acutely deconditioned men and women (Greenleaf et al., 1998; Stenger et al., 2007, 2012; Evans et al., 2015; Goswami et al., 2015b; Zhang et al., 2017). In studies conducted before 2007, we looked at effects of AG training (45 min a day, 5 days a week, over a period of 3 weeks) with respect to the ability of short bouts of AG to improve orthostatic tolerance over ambulatory, pretraining tolerance. We found the significant increase in tolerance after AG training to be associated with decreased resting blood pressure and vascular resistance and increased stroke volume (Greenleaf et al., 1998; Evans et al., 2004; Stenger et al., 2007). The improvement in vascular responsiveness was demonstrated through increased low frequency spectral power of blood pressure and heart rate as well as a doubling of the norepinephrine response during tilt (Greenleaf et al., 1998; Evans et al., 2004; Stenger et al., 2007).

#### Gender Differences in Ambulatory Responses to Chronic AG Exposure

Similar to tolerance for Earth gravity or matched levels of LBNP, women clearly demonstrate a lower tolerance for orthostatic stress than do men (Ludwig et al., 1987; Frey and Hoffler, 1998; Evans et al., 2004; Fong et al., 2007; Stenger et al., 2007; Opatz et al., 2014). In our studies, 3 weeks of AG training (45 min/day, 5 days a week) improved ambulatory men’s orthostatic tolerance more than it improved ambulatory women’s tolerance and exercise during AG improved tolerance more than did passive AG. An improvement in orthostatic tolerance was not seen for these ambulatory women unless exercise accompanied the AG sessions (Evans et al., 2004; Stenger et al., 2007). Other investigators determined that a primary source of the increased protection against OI provided by AG training, resulted from an increased ability to mobilize stroke volume and cardiac output during orthostatic stress that was more evident in men than women (Convertino et al., 1998).

In a test of brain cortical activation during stepped increases in G load (to presyncope), beta wave (12.5–35 Hz) activity increased in both men and women, while alpha wave (7.5–12.5 Hz) activity increased only in men (Schneider et al., 2014). This sex difference in cortical activation in response to increasing levels of AG may have implications in the observed sex differences in cardiovascular responses to AG (Stenger et al., 2012).

### Deconditioned Subjects

Recently, Clement and Pavy-Le Traon (2004) and Clement et al. (2016), reviewed studies that explored AG as a countermeasure to deconditioning evoked by bed rest and dry immersion. Results from the 18 studies reviewed, all males, indicated that AG, applied over as little as 30 min twice a day (Sasaki et al., 1999) was successful at mitigating OI in men who were deconditioned by these protocols. Other results included reductions in exaggerated responses to orthostasis, maintenance of autonomic cardiovascular function and attenuation of plasma volume loss. One review also indicated that AG applied intermittently rather than being held at a constant value, was better tolerated by men (Clement et al., 2016). However, the spectrum of AG effects on deconditioned men and women will not be definitive until AG has been tested in deconditioned women.

#### Acute Exposure to AG

##### Hypovolemic men

Recently, we observed that 90 min of AG exposure in a protocol individualized to provide a common stimulus to each person, improved the orthostatic tolerance limit of hypovolemic men compared to a day in which the same men had been mildly deconditioned by hypovolemia plus 90 min of 6 degree HDBR (Evans et al., 2015). On both study days, subjects had undergone a diet and furosemide protocol to reduce their plasma volume to match the reduction observed during spaceflight. In a companion study, we found that applying a similar, individualized, AG protocol to a separate group of *normovolemic* men significantly improved the OTL of that group compared to a day in which the same subjects had rested supine for 90 min (Goswami et al., 2015b). Both studies determined that a 90 min exposure to increasing levels of AG (to presyncope) improved a subsequent test of their OTL by up to 30% (hypovolemic men) compared to their OTL on the supine/HDBR day. Mechanisms for improvement in men’s orthostatic tolerance appeared to be a result of decreased resting blood pressure accompanied by increased cardiac output at rest and during orthostatic testing following AG exposure (Evans et al., 2015). The Goswami et al. (2015b) study similarly found decreased blood pressure but did not find increased cardiac output in men following their exposure to AG.

In the Clement review of AG as a countermeasure to deconditioning, 18 studies of men undergoing HDBR or dry immersion reported that acute AG applied intermittently was effective to improve orthostatic tolerance, increase MSNA, maintain exercise capacity, and reduce exaggerated responses to orthostatic testing, but was not effective to return plasma volume to normal (Clement et al., 2016).

##### Hypovolemic women

The hypovolemic study above included women. These women’s OTL was improved by the 90 min exposure to AG compared to the day on which they were exposed to 90 min of 6 degree HDBR (Evans et al., 2015). As with normovolemic women and men and hypovolemic men, the mechanism of OTL improvement in hypovolemic women was primarily through increased resting cardiac output (Evans et al., 2015; Goswami et al., 2015b).

An additional report of autonomic responses to the AG exposure of hypovolemic men and women of the Evans study (above) indicated that mechanisms of improved OTL following AG exposure, also included increased responsiveness of the cardiac baroreflex to orthostatic stress (both men and women) (Zhang et al., 2017).

##### Gender perspectives in deconditioned subjects’ responses to acute AG

In addition to the effects of AG to improve baroreflex responsiveness in men and women noted above (Zhang et al., 2017), we determined that AG exposure increased men’s low frequency spectral power of systolic blood pressure during the subsequent test of their OTL but did not change women’s. In that study we also determined that men’s resting blood pressure declined after exposure to AG, but women’s blood pressure was not different on the 2 days either at rest or during orthostatic tolerance testing (Evans et al., 2015). Different between the Evans et al. (2015) and Goswami et al. (2015b) studies was the increase in women’s cardiac output which was significantly greater on the AG day for women separately in the Goswami study but only as part of the whole group in the Evans study. Similarities between men and women in this study included increased orthostatic tolerance (Evans et al., 2015)and improved baroreflex activity (Zhang et al., 2017) on the day subjects had previously been exposed to AG. Major differences between men and women consisted of decreased blood pressure (Evans et al., 2015) and increased low frequency spectral power of blood pressure in men but not women following exposure to AG (Zhang et al., 2017).

#### Chronic Exposure to AG

Studies have been performed to determine effects of AG applied periodically to men during bed rest deconditioning (White et al., 1966; Iwasaki et al., 2001; Pavy-Le Traon et al., 2007; Stenger et al., 2012; Linnarsson et al., 2015). White et al. (1966) used 1.75 Gz (heart level) applied in four, 20 min daily sessions, to prevent the development of OI in men during 10 days of bed rest. Pavy-Le Traon et al.’s (2007) review of 20 years of bed rest studies, included studies in which gravity was used as a countermeasure; it was apparent from early days, that as little as standing 2 h a day would lessen the incidence of OI in men bed rested for 4 days (Vernikos et al., 1996). Iwasaki et al. (2001) study determined that two, 30 min bouts of 2 Gz applied daily, could prevent the development of OI as well as shifts in autonomic balance toward sympathetic dominance during 4 days of HDBR. In 2006, we participated in the first NASA study to survey a comprehensive physiologic response to 3 weeks of HDBR in a group of men who received an hour of AG per day (+1 Gz at the heart) and compared those results to a control group of men who did not undergo the AG exposure (Stenger et al., 2012). In the group of men who received AG, the HDBR-induced decreases in orthostatic tolerance and maximum oxygen consumption were significantly smaller and the profiles of vasoactive hormones in response to head up tilt were improved in the AG group compared to the control group. The recent study by Linnarsson et al. (2015) was able to establish that an intermittent (6 each, 5 min exposures to AG) protocol applied daily to men undergoing 5 days of HDBR resulted in orthostatic tolerance nearer to that of pre-HDBR than did a protocol that delivered 30 min of continuous AG to the same men.

##### Gender perspectives in deconditioned subject’s responses to chronic AG

We could not find any studies that determined women’s cardiovascular responses to chronic AG exposure during bed rest, HDBR, or immersion deconditioning. Our group’s study of acute exposure to AG following furosemide infusion to induce plasma volume loss similar to that of spaceflight is the only study from which we are able to compare deconditioned men’s and women’s responses before and after AG (Evans et al., 2015). Similarities between these deconditioned men and women included improved orthostatic tolerance (Evans et al., 2015) and baroreflex function (Zhang et al., 2017) on the day subjects had been exposed to AG. Major differences between men and women consisted of decreased blood pressure (Evans et al., 2015) and increased low frequency spectral power of blood pressure in men but not women following exposure to AG (Zhang et al., 2017).

## Conclusion and Perspectives

There is emerging evidence that an individual-specific AG training protocol may be a useful tool to assess orthostatic tolerance in both males and females. This has been verified in both normovolemic and hypovolemic men and women. Future studies should consider the usage of individual-specific AG training as highlighted in the Evans et al. (2015) and Goswami et al. (2015b) studies.

Data from the above studies as well as directions from NASA and ESA administrators, indicate that cardiovascular deconditioning as a result of space flight may not be overcome by current countermeasures [Chapter 7 of Recapturing a Future for Space Exploration (National Research Council, 2011)]. Gaps in knowledge include whether AG applied to astronauts will preserve physiologic systems’ (including the cardiovascular system) integrity so that astronauts can return safely to live on Earth. There is a cascade of basic studies that will follow the addition of an AG facility to other space-based facilities in order that future investigators be able to ask and answer basic questions as to the protocols that will render space fight safe and will introduce new hardware for future study. For those reasons, the “Crosscutting Issues for Humans in the Space Environment” document recommended that “NASA should reinitiate a vigorous program to…develop a simple short-radius human centrifuge for eventual evaluation experiments aboard the ISS.”

## Author Contributions

JE wrote this review based upon many years of collaborative research into the cardiovascular effects of gravity. CK contributed to this review based upon many years of collaborative research into the cardiovascular effects of gravity. NG contributed to this review based upon his collaborative research into the cardiovascular effects of gravity.

## Conflict of Interest Statement

The authors declare that the research was conducted in the absence of any commercial or financial relationships that could be construed as a potential conflict of interest.
